# In Vitro and In Vivo Antimelanogenesis Effects of Leaf Essential Oil from *Agathis dammara*

**DOI:** 10.3390/pharmaceutics15092269

**Published:** 2023-09-02

**Authors:** Yu-Tung Ho, I-Hsuan Liu, Shang-Tzen Chang, Sheng-Yang Wang, Hui-Ting Chang

**Affiliations:** 1 School of Forestry and Resource Conservation, National Taiwan University, Taipei 10617, Taiwan; r10625032@ntu.edu.tw (Y.-T.H.); peter@ntu.edu.tw (S.-T.C.); 2 Department of Animal Science and Technology, National Taiwan University, Taipei 10617, Taiwan; ihliu@ntu.edu.tw; 3 Department of Forestry, National Chung Hsing University, Taichung 40227, Taiwan; taiwanfir@dragon.nchu.edu.tw; 4 Special Crop and Metabolome Discipline Cluster, Academy Circle Economy, National Chung Hsing University, Taichung 40227, Taiwan

**Keywords:** *Agathis dammara*, antimelanogenesis effect, antityrosinase activity, essential oil, melanin, zebrafish (*Danio rerio*)

## Abstract

*Agathis* species are widely distributed around Southeast Asia, Australasia, South Pacific islands, and etc. Traditionally, *Agathis* species have been used as the folk medicines, the common ethnopharmacological uses of *Agathis* genus are the treatments of headache and myalgia. This study aims to investigate the chemical composition of *Agathis dammara* (Lamb.) Rich. leaf essential oil and to explore its antimelanogenesis effect. The chemical constituents of leaf essential oil are analyzed using gas chromatography-mass spectrometry (GC-MS), the major constituents of leaf essential oil are sesquiterpenoids. The major constituents are δ-cadinene (16.12%), followed by γ-gurjunene (15.57%), 16-kaurene (12.43%), β-caryophyllene (8.58%), germacrene D (8.53%), and γ-cadinene (5.33%). As for the in vitro antityrosinase activity, leaf essential oil inhibit the tyrosinase activity of mushroom when the substrate is 3,4-dihydroxyphenylalanine (L-DOPA). Leaf essential oil prevents tyrosinase from acting as diphenolase and catalyzing L-DOPA to dopaquinone, and converting into dark melanin pigments. *A. dammara* leaf essential oil also exhibits the in vivo antimelanogenesis effect, leaf essential oil reduces 43.48% of melanin formation in zebrafish embryos at the concentration of 50 μg/mL. Results reveal *A. dammara* leaf essential oil has the potential for developing the skin whitening drug and depigmentation ingredient for hyperpigmentary disorders.

## 1. Introduction

Araucariaceae is an ancient family and widespread in the Jurassic period, distribution in both hemispheres including numerous taxa. Araucariaceae species are present in South America, Australasia, South Pacific islands, Southeast Asia and etc. Araucariaceae family contains four genera, including *Agathis*, *Araucaria*, *Columbea*, and *Wollemia*, com-monly known as kauri or dammar. The ethnopharmacological uses of the Araucariaceae family are versatile and numerous, including emollient and antiseptic properties, and to treat headache, myalgia, respiratory infections, rheumatisms, skin wounds, insomnia, and so on [1,2,3,4,5].

Genus *Agathis* are widely distributed in Australia, New Zealand, South Pacific islands, and Southeast Asia, the species are characterized by their brownish black bark, leathery and ovate-lanceolate leaves, circular branch scars on their trunks, and their milky resins. *Agathis* genus contains 18 accepted species and 4 unresolved species [1,2,3,4]. To date, the timbers of *Agathis* spp. are recognized as good materials for processing, the timber is straight-grained, heavier and harder wood, knot free, with a fine textured and lustrous surface [3]. Gum copal is harvested by tapping or cutting the living *Agathis* trees. Resins and gums from *Agathis* spp. have been used as varnishes, lacquers, adhesive, and etc. [1,5]. According to the related research reports, α-pinene, β-pinene, camphene, α-copaene, α-cubebene, β-caryophyllene, δ-cadinene, *allo*-aromadendrene, aromadendrene, germacrene D, limonene, myrcene, sabinene, spathulenol, and 16-kaurene are the most abundant constituents found in the essential oils of *Agathis* species [2,3]. The biological activities of genus *Agathis* include antibacterial, antifungal, anti-inflammatory, antileishmanial, antiplasmodial, cytotoxic activities, etc. in literatures [3,5,6,7,8]. Verma et al. investigated the antibacterial activity of resin essential oil from *A. robusta* (C. Moore ex F. Muell.) Bailey (Queensland Kauri), resin essential oil showed moderate activity against *Staphylococcus epidermidis* and good activity against *S. aureus* in the disc diffusion assay [5]. The leaf essential oil of *A. dammara* (Lamb.) Rich. was also found to have significant antibacterial activity against *Staphylococcus aureus* (Gram positive bacterium) and *Pseudomonas aeruginosa* (Gram negative bacterium) in the disc diffusion method and microwell dilution assay [6]. *A. atropurpurea* B. Hyland bark resin exhibited effective antileishmanial activity against *Leishmania amazonensis* promastigotes and amastigotes [7]. Resins from *A. atropurpurea* showed slight antifungal activity against *Aspergillus niger* and *Rhizopus stolonifera*, which are the important and widespread human and agricultural harmful pathogens [8].

Melanin, the products of melanogenesis, is produced in melanocytes and stored in melanosomes. Normally, melanin prevents skin from ultraviolet (UV) damage, photocarcinogenesis, and interference of vitamin D3 formation [9,10,11,12]. However, abnormal melanin production and accumulation could induce hyperpigmentation (age spots, freckles and melasma), and melanoma, the skin tumor [9,13,14,15]. In order to prevent from hyperpigmentation, suppressing tyrosinase activity is one of the most effective strategies. Tyrosinase (EC 1.14.18.1), a copper containing enzyme, is a key enzyme in melanogenesis. The site of copper ion pair (central domain) coordinating with 6 histidine residues formed an active site of tyrosinase [9,16,17]. Depending on the substrates binding to the active site, tyrosinase catalyzes two reactions. When tyrosinase binds with L-tyrosine (monophenol), it acts as monophenolase and helps hydroxylation of L-tyrosine. When tyrosinase (diphenolase) binds with 3,4-dihydroxyphenylalanine (L-DOPA), further oxidizes of L-DOPA to dopaquinone occurs, and leads to the formation of eumelanin and pheomelanin [16,17,18].

Currently, the well-known ingredients, hydroquinone, kojic acid, ascorbic acid, and arbutin, prevent L-tyrosine or L-DOPA converting to dopaquinone by inhibiting tyrosinase activity [17,19,20]. These compounds may bring about some disadvantages. For example, hydroquinone could cause skin irritation and cell mutation; kojic acid is carcinogenic; ascorbic acid and arbutin has poor storability [14,17,21,22,23]. Due to the drawbacks and obstacles, alternatives from natural sources have been increasingly researched [24,25,26,27,28,29]. *Elaeocarpus serratus* Linn. (Tiliaceae) leaf extract and its active compounds, gallic acid, myricetin and mearnsetin, present tyrosinase inhibitory activity and antimelanogenesis effect on zebrafish (*Danio rerio*) embryos [25]. Tetrahydrocurcumin, 1,7-bis(4-hydroxy-3-methoxyphenyl)heptane-3,5-dione, is the metabolite of curcumin, derived from the *Curcuma longa* L. rhizome. Tetrahydrocurcumin can inhibit the melanin production in B16F10 melanoma cells induced by melanocyte-stimulating hormone (α-MSH), and modulate the expressions of important cellular enzymes, tyrosinase, tyrosinase-related protein 1 (TRP-1), and tyrosinase-related protein 2 (TRP-2), which involved the biosynthetic production of melanin [26]. Green tea (*Camellia sinensis* (L.) Kuntze) extract and its constituents, including gallocatechin-3-*O*-gallate (GCG), epigallocatechin-3-*O*-gallate (EGCG), and epicatechin-3-*O*-gallate (ECG) exhibited the effective antityrosinase efficacy [27]. Several plant natural products were reported to possess the antityrosinase activity and antimelanogenesis effect, including citrus essential oils, sesamol, haginin A, and etc. [28,29]. Bioactive ginsenosides, including ginsenoside Rh6, vina-ginsenoside R4, vina-ginsenoside R13, ginsenoside Rh23, and floralginsenoside A, isolated from *Panax ginseng* C. A. Meyer (Araliaceae) leaf and berry exhibit the antimelanogenesis effect in zebrafish model [30,31,32]. The aims of the study are to investigate the chemical composition and antimelanogenesis effects (in vitro and in vivo) of *A. dammara* leaf essential oil.

## 2. Materials and Methods

### 2.1. Plant Material

The fresh and mature leaves (dark green) of *Agathis dammara* (Lamb.) Rich. were collected from the campus (25°01′03.0″ N 121°32′21.1″ E) of National Taiwan University, Taipei, Taiwan in March 2022. The diameter at breast height (DBH) of the tree is 31.9 cm, and the height is 11.6 m. The species was identified by Dr. Chih-Chieh Yu, Xishuangbanna Tropical Botanical Garden, Chinese Academy of Sciences. Voucher specimen (AD0322) has been kept in the laboratory of Chemical Utilization of Biomaterials, School of Forestry and Resource Conservation, National Taiwan University.

### 2.2. Hydrodistillation of Essential Oil

The fresh and mature leaves of *A. dammara* were hydrodistilled for 6 h by using the Clevenger apparatus. After the hydrodistillation, the leaf essential oil was placed in a dark glass bottle and stored in a refrigerator at 4 °C for further investigation [33,34,35].

### 2.3. Gas Chromatography-Mass Spectrometry (GC-MS) Analysis

To investigate the chemical composition of the essential oil, the analysis of *A. dammara* leaf oil was carried out on a Trace GC Ultra (Thermo Fisher Scientific, Waltham, MA, USA) equipped with a DB-5 MS column (Crossbond 5% methylpolysiloxane, 30.0 m length × 0.25 mm diameter, thickness 0.25 μm; Agilent Technologies, Palo Alto, CA, USA). The oven temperature started from 60 °C for 3 min, programed at 3 °C/min to 120 °C, 5 °C/min to 240 °C for 3 min. The injector temperature was held at 250 °C, and split ratio was 10:1. The carrier gas was helium; 1.0 mL/min flow rate; ion source temperature 250 °C; mass range 50–650 amu. The constituents of essential oil were characterized by National Institute of Standards and Technology (NIST) V.2.0 and Wiley 7.0 GC-MS libraries and Kovats indexes in the reference [36]. Kovats indexes of the constituents are determined by retention times of *n*-alkanes (C7–C30) on the DB-5MS column. The relative contents of constituents were determined by integrating the peaks on total ion chromatograms (TIC) [37,38].

### 2.4. Antityrosinase Assay

Tyrosinase is an essential enzyme in melanogenesis. The antimelanogeneic effect of specimen can be preliminarily determined by its tyrosinase inhibitory activity. The activity was determined by previous assays with minor modification [24,39]. The specimen-buffer mixture (110 μL) was added in a 96 well microplate. The specimen was diluted in DMSO and mixed with 0.1 M potassium phosphate buffer (pH = 6.8). Kojic acid (Sigma, St. Louis, MO, USA) and arbutin (Sigma, St. Louis, MO, USA) were used as the positive controls. Subsequently, 50 μL of 200 U/mL mushroom tyrosinase (EC 1.14.18.1; Sigma, St. Louis, MO, USA) was added and mixed with the specimen-buffer mixture. After mixed with 40 μL of substrate (L-tyrosine and L-DOPA), the mixture was incubated for 10 min at room temperature. Next, the 96-well microplate was sent into SPEOTROstar, the microplate reader (BMG Labtech, Ortenberg, Germany), and the absorbance (475 nm) of each well was measured. The tyrosinase inhibitory activity was presented as inhibition rate and half maximal inhibitory concentration (IC_50_). The equation of inhibition rate is as followed: Inhibition (%) = [1 − (A_specimen_ − A_specimen’s blank_)/(A_control_ − A_control’s blank_)] × 100. The IC_50_ was determined by concentration-response curve. The experiments were performed in triplicate.

### 2.5. Antimelanogenesis Effect in Zebrafish Embryos

Wild-type AB zebrafish (*Danio rerio*) were maintained under synchronized conditions (26–30 °C; pH = 6.5–7.5; 14 h/10 h of light/dark cycle). 9 h-post-fertilization (hpf) embryos were collected and arrayed in 24 well plate. Each well (1 mL) contains 5 embryos, E3 medium, and specimen diluted in 1% DMSO. After that, the plate was incubated at 28 °C for 48 h, and the 57 hpf zebrafish embryo were photographed under a stereomicroscope (Hamlet SEM-H, Taiwan) at 40× magnification. The melanin content of zebrafish embryos was quantified by ImageJ (V1.52a) and presented as inhibition rate. The calculation of inhibition is: Inhibition (%) = [1 − (Intensity _specimen_/Intensity _control_)] × 100. 1-Phenyl-2-thiourea (PTU), kojic acid, and arbutin were the positive controls [24,27,40]. The protocol was approved by the Institutional Animal Care and Use Committee (IACUC) in National Taiwan University (IACUC Approval No: NTU-112-EL-00072), all zebrafish were handled by the 3R’s principles of laboratory animal care and use.

### 2.6. Statistical Analysis

The statistical analysis of data obtained in the study was analyzed by SPSS (Statistical Product and Service Solutions) (Chicago, IL, USA) Version 16 with the Scheffe’s multiple comparison test, a *post-hoc* multiple comparison method. The confidence interval was set at the level of 95%.

## 3. Results and Discussion

### 3.1. Chemical Constituents of A. dammara Leaf Essential Oil

**Figure 1:**
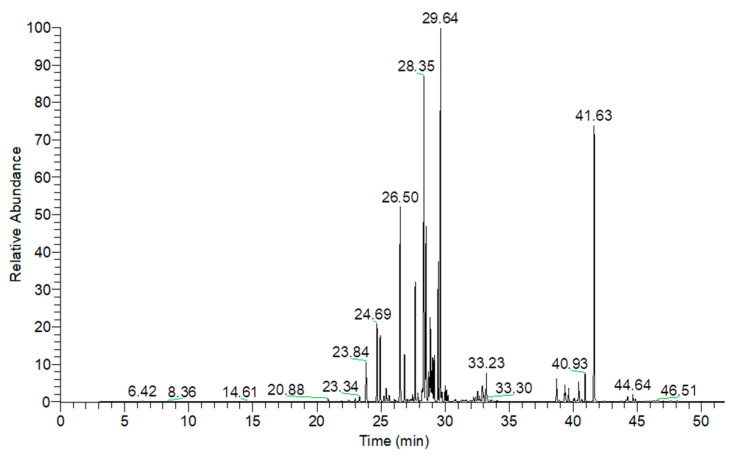
Gas chromatogram of *Agathis dammara* leaf essential oil.

**Figure 2:**
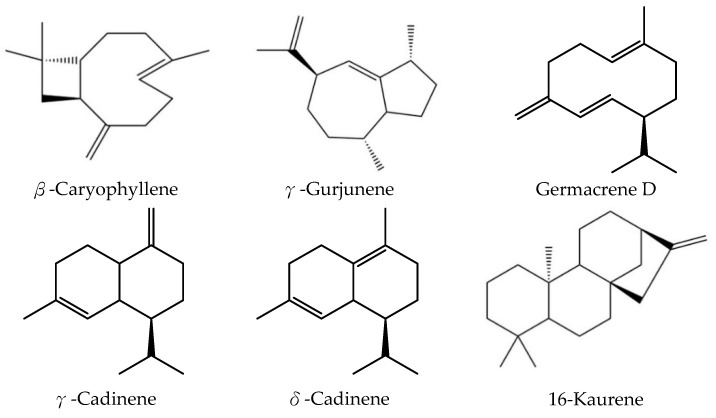
Chemical structures of major constituents of *A. dammara* leaf essential oil.

In this study, leaf essential oil was hydrodistilled from the mature dark green leaves of *A. dammara*. The yield of leaf essential oil was 0.15 ± 0.05%, on dry matter basis of leaf. Figure 1 showed the gas chromatogram of *A. dammara* leaf essential oil. The constituents of *A. dammara* leaf essential oil were analyzed by using GC-MS, and there are 21 constituents found in the chromatogram. According to Table 1, the leaf essential oil contained sesquiterpene hydrocarbons (69.35 ± 2.18%), diterpene hydrocarbons (13.64 ± 1.46%), and oxygenated sesquiterpenes (3.10 ± 0.21%). Except for 16-kaurene (12.43 ± 1.32%), most of the major constituents were sesquiterpenoids, including δ-cadinene (16.12 ± 0.53%), γ-gurjunene (15.57 ± 0.49%), β-caryophyllene (8.58 ± 0.94%), germacrene D (8.53 ± 0.20%), and γ-cadinene (5.33 ± 0.16%) (Figure 2). Chen et al. analyzed the chemical constituents of *A. dammara* leaf essential oil, the major components were limonene (36.81%), β-bisabolene (33.43%) and β-myrcene (25.48%) [6]; the content of monoterpenoids, limonene and β-myrcene, was over 50%. The result of the study is inconsistent with our analysis of the chemical compositions of leaf essential oil. New or young leaves of *A. dammara* are light green color, later turn dark green after a few days, and keep dark green color. Differences between two studies of chemical constituents of leaf essential oil may be due to the maturity of leaf, collected region, environment temperature, sunlight, season, and etc.

The sesquiterpenoids of leaf essential oil were dominated by compounds with cadinane skeleton, including α-muurolene, α-cadinene, γ-cadinene, δ-cadinene, *trans*-1,4-cadinadiene, α-calacorene, β-calacorene, 1-epi-cubenol, α-cadinol, and δ-cadinol. The compounds with copane skeleton were α-copaene and α-ylangene. α-Cubebene and β-cubebene were classified as compounds with cubebane skeleton. α-Caryophyllene and β-caryophyllene were characterized as caryophyllane skeleton. Other compounds, δ-elemene, γ-gurjunene, and germacrene D, were characterized as elemane skeleton, guaiane skeleton, and germacrene skeleton, respectively. As for the diterpenes, rimuene and 16-kaurene, they were classified as rosane and kaurane skeleton compounds.

The major constituents of *A. dammara* leaf essential oil were likewise observed in those of *A. robusta* F. M. Bailey and *A. macrophylla* (Lindl.) Mast., which share a same clade with *A. dammara* in the phylogenetic tree [41,42]. Brophy et al. reported that constituents of *A. robusta* leaf essential oil were spathulenol (36.7%), rimuene (5.6%), α-pinene (3.7%), caryophyllene oxide (3.1%), δ-cadinene (1.6%), β-caryophyllene (1.4%), β-pinene (1.2%), and germacrene D (1.1%) [2]. Verma et al. also reported the chemical constituents of *A. robusta* leaf essential oil was predominated by sesquiterpenoids (75.6%), containing β-selinene (18.1%), caryophyllene oxide (11.5%), spathulenol (10.5%), α-selinene (9.8%), γ -muurolene (5.8%), and etc.; the leaf essential oil also contained diterpenoid, rimuene (14.2%, second major component) [5]. The main constituents of *A. atropurpurea* the principal components were the monoterpene α-pinene (7.9%), the sesquiterpenes δ-cadinene (9.0%) and the diterpenes phyllocladene (12.5%) and 16-kaurene (19.4%) [2]. The identified constituents of *A. dammara* leaf essential oil were somewhat similar to those of of *A. robusta, A. macrophylla*, and *A. atropurpurea* leaf essential oils.

### 3.2. Tyrosinase Inhibitory Activity of A. dammara Leaf Essential Oil

Tyrosinase which catalyzes L-tyrosine and L-DOPA into dopaquinone and dopachrome, respectively, is the essential enzyme in melanogenesis. To inhibit the melanogenesis process, suppressing the tyrosinase activity is one of the most common and effective strategies [43].

**Figure 3:**
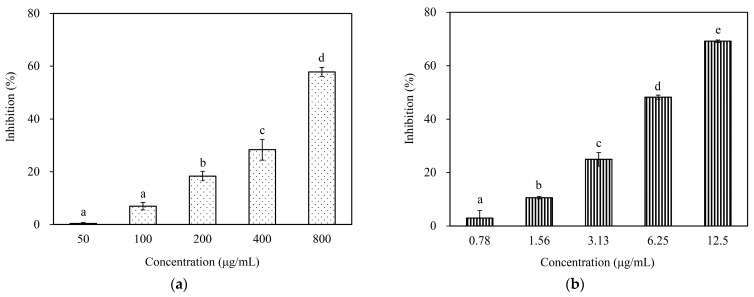
Mushroom tyrosinase inhibitory activity of *A. dammara* leaf essential oil. When using L-DOPA as the substrate. (**a**) *A. dammara* leaf essential oil; (**b**) kojic acid. Different letters (a–d; a–e) represent significantly different at the level of *p* < 0.05 according to the Scheffe’s test.

Figure 3 shows that leaf essential oil could prevent tyrosinase from acting as diphenolase and catalyzing L-DOPA to dopaquinone. Tyrosinase inhibitory activity of *A. dammara* leaf essential oil presented the dose-dependent effect. The inhibition rates were 6.93 ± 1.37%, 18.36 ± 1.82%, and 28.38 ± 3.95% at the concentration of 100, 200, and 400 μg/mL, respectively. Kojic acid, positive control, presented inhibition of diphenolase. Kojic acid showed 69.15% inhibition at the concentration of 12.5 μg/mL, followed by 48.20%, 24.94%, 10.57% at the concentrations of 6.25, 3.13, and 1.56 μg/mL. The IC_50_ values of leaf essential oil and kojic acid were 690.02 ± 18.85 and 6.74 ± 0.09 μg/mL, respectively (Table 2). When the substrate was L-tyrosine, no significant inhibitory activity of leaf essential oil was observed. As for positive control, kojic acid (IC_50_ = 2.46 ± 0.06 μg/mL) presented the tyrosinase inhibitory activity (Table 2).

Huang et al. examined the tyrosinase inhibitory activity of *Vitex negundo* Linn. (Lamiaceae) leaf essential oil, the inhibition rate of leaf essential oil against tyrosinase were 26.6% at the concentration of 5 mg/mL, using L-DOPA as the substrate [44]. Cheraif et al. evaluated the antityrosinase activity of essential oils from six Algerian plants, *Juniperus oxycedrus* L. (Cupressaceae) essential oil displayed the best tyrosinase inhibitory activity with a tyrosinase inhibition rate of 39.65% at the concentration of 1 mg/mL [45]. *Etlingera elatior* (Jack) R. M. Sm. (Zingiberaceae) leaf essential oil exhibited a moderate activity against tyrosinase, with an IC_50_ value of 2.34 ± 0.04 mg/mL when using L-DOPA as the substrate [46]. Salleh et al. investigated the antityrosinase effects of leaf essential oil and bark essential oil of *Beilschmiedia madang* Blume (Lauraceae), using L-DOPA as the substrate, inhibition rates of leaf essential oil and bark essential oil were 53.1 ± 0.2% and 51.2 ± 0.2%, respectively, at the concentration of 1 mg/mL [47]. *Pogostemon plectranthoides* Desf. (Lamiaceae) leaf essential oils collected from three bio-geographical regions also had the antityrosinase activity. Leaf essential oils were rich in sesquiterpenes, and the active leaf essential oil possessed the tyrosinase inhibitory effect with the inhibition rate of ca. 50% at the concentration of 1 mg/mL, using L-DOPA as the substrate [48]. These related researches illustrated that *A. dammara* leaf essential oil exhibited the tyrosinase inhibition potential.

### 3.3. Antimelanogenesis Effect of A. dammara Leaf Essential Oil in Zebrafish

**Figure 4:**
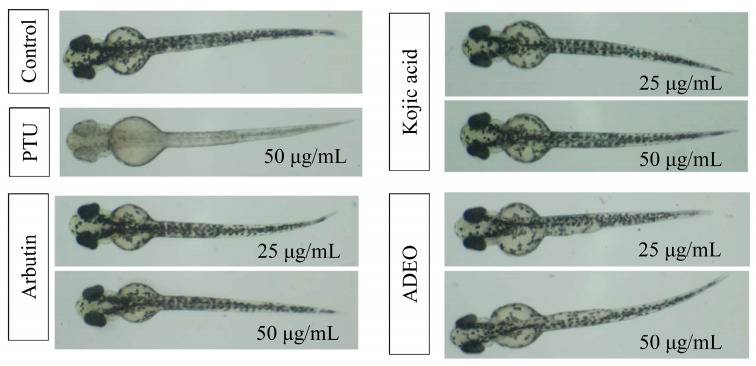
Effects of specimens on melanogenesis of zebrafish embryos. PTU: 1-Phenyl-2-thiourea; ADEO: *A. dammara* leaf essential oil.

Zebrafish (*D. rerio*) has been a powerful and wide used model organism and the efficient alternative to other animal models in multiple researches. In the present study, zebrafish is a suitable vertebrate model for melanogenesis study because of its easily observed pigmentation and the similarity of gene sequences and organ systems [21,49,50]. The antimelanogenesis effects in the zebrafish model caused by different treatments were shown in Figure 4. In control group, the patterns of pigmentation presented normal dispersion. After treated with *A. dammara* leaf essential oil, kojic acid, and arbutin, the zebrafish embryos displayed less amount of pigmentation. As for PTU treatment, the zebrafish embryos became transparent after 48 h incubation, inhibition rate of melanin was 98.23 ± 1.01% at the concentration of 50 μg/mL.

Table 3 shows the inhibitory effect of each treatment on melanogenesis of zebrafish embryo, including leaf essential oil, arbutin, kojic acid, and PTU. The melanin contents of zebrafish embryos could decrease by 21.03%, 37.36% and 43.48% with treatment of leaf essential oil at a concentration of 12.5, 25 and 50 μg/mL, with a dose-dependent manner. In comparison with the treatment of arbutin, leaf essential oil presented higher antimelanogenesis effect at the concentrations of 25 and 50 μg/mL. Significant statistical differences (*p* < 0.05) were observed between the treatment of leaf essential oil (43.48%) and the treatment of arbutin (21.49%) at the concentration of 50 μg/mL. Another positive control, kojic acid, inhibited 18.53% and 20.44% of melanin production of zebrafish embryos at the concentrations of 25 and 50 μg/mL, respectively. Kojic acid exhibited the lower melanin inhibition in zebrafish embryos than leaf essential oil treatments did.

Chelly et al. (2021) evaluated the antimelanogenesis activities of methanolic extracts of *Rhanterium suaveolens* Desf. (Asteraceae) flower, stem, and leaf, with in vitro antityrosinase inhibition assay and in vivo zebrafish embryo assay [51]. Among these part extracts of *R. suaveolens*, flower extract exhibited the best antityrosinase effect on tyrosinase induced L-DOPA oxidation. Flower extract also possessed the statistically significant decrease of melanin formation in zebrafish embryo at the concentrations of 0.5 and 1 mg/mL. The reduction of melanin formation in zebrafish embryo was less than 50% after 48 h treatment of 1 mg/mL concentration of *R. suaveolens* flower extract. *Dalbergia pinnata* (Lour.) Prain essential oil inhibited 18.77% of melanin production of zebrafish embryos at a concentration of 30 μg/mL [52]. *Bletilla striata* (Thunb.) Rchb. f. tuber extract reduced 18.35% and 24.39% of melanin formation of zebrafish embryos at the concentrations of 10 μg/mL and 30 μg/mL, respectively [53]. The results in this study revealed that *A. dammara* leaf essential oil possessed a potent melanogenesis inhibition activity in zebrafish embryos.

Yang et al. investigated the antimelanogenesis effect of lime mint (*Mentha aquatica* × *M. suaveolens*) essential oil and its main constituents in B16F10 murine melanoma cells. The main constituents of lime mint essential oil were D-limonene (41.10%), D-carvone (8.58%), δ-selinene (6.73%), and β-caryophyllene (6.24%). Among these main constituents, only β-caryophyllene exhibited the antimelanogenesis activity in a dose dependent manner. Results revealed that β-caryophyllene can modulate the expression of melanogenesis related proteins, including tyrosinase, microphthalmia-associated transcription factor (MITF), TRP-1, and TRP-2. β-Caryophyllene reduces cellular melanogenesis by down regulating the expression of these melanogenesis related proteins, resulting in the decrease in melanin content of B16F10 murine melanoma cells at the concentration of 150 μM [54]. According this related research, compound β-caryophyllene might contribute the melanogenesis effect of *A. dammar* leaf essential oil.

## 4. Conclusions

For the assessment of antimelanogenesis effect of essential oil from *A. dammara* leaf, both in vitro and in vivo antimelanogenesis assays were conducted in this study. The chemical constituents of *A. dammara* leaf essential oil was dominated by sesquiterpenoids through the GC-MS analysis. The most abundant constituent was δ-cadinene (16.12%), followed by γ-gurjunene (15.57%), 16-kaurene (12.43%), β-caryophyllene (8.58%), germacrene D (8.53%), and γ-cadinene (5.33%). Among all the constituents, most of the sesquiterpenoids were the compounds with cadinane skeleton. In vitro antityrosinase assay, *A. dammara* leaf essential oil showed diphenolase inhibitory activity. When the substrate was L-DOPA, the IC_50_ value of leaf essential oil was 690.02 μg/mL. According to the in vivo zebrafish embryo assay, leaf essential oil has shown efficacy to reduce the melanin formation in zebrafish embryos. At a concentration of 50 μg/mL, leaf essential oil, arbutin, and kojic acid inhibited 43.48%, 21.49%, and 20.44%, respectively, of melanin production of zebrafish embryos. Antimelanogenesis activity of leaf essential oil was better than those of arbutin and kojic acid in zebrafish embryos. These results revealed *A. dammara* leaf essential oil has in vitro and in vivo antimelanogenesis activities, and has the potential to be the skin whitening drug and depigmentation ingredient for hyperpigmentary disorders.

## Figures and Tables

**Table 1:** Constituents of essential oil from *Agathis dammara* leaves.

RT ^a^ (min)	KI ^b^	rKI ^c^	Constituent	M.F.	M.W.	Relative Content (%)	Identified Method ^d^
23.34	1335	1338	δ-Elemene	C_15_H_24_	204	0.23 ± 0.03	MS, KI
23.84	1348	1351	α-Cubebene	C_15_H_24_	204	1.65 ± 0.21	MS, KI
24.69	1369	1371	α-Ylangene	C_15_H_24_	204	2.84 ± 0.39	MS, KI
24.94	1375	1376	α-Copaene	C_15_H_24_	204	2.47 ± 0.31	MS, KI
25.40	1386	1388	β-Cubebene	C_15_H_24_	204	0.62 ± 0.06	MS, KI
26.50	1416	1419	β-Caryophyllene	C_15_H_24_	204	8.58 ± 0.94	MS, KI
27.68	1454	1454	α-Caryophyllene	C_15_H_24_	204	4.37 ± 0.31	MS, KI
28.35	1475	1477	γ-Gurjunene	C_15_H_24_	204	15.57 ± 0.49	MS, KI
28.50	1479	1481	Germacrene D	C_15_H_24_	204	8.53 ± 0.20	MS, KI
29.03	1495	1500	α-Muurolene	C_15_H_24_	204	1.71 ± 0.01	MS, KI
29.46	1510	1513	γ-Cadinene	C_15_H_24_	204	5.33 ± 0.16	MS, KI
29.64	1517	1522	δ-Cadinene	C_15_H_24_	204	16.12 ± 0.53	MS, KI
30.00	1531	1534	*trans*-1,4-Cadinadiene	C_15_H_24_	204	0.58 ± 0.03	MS, KI
30.12	1535	1538	α-Cadinene	C_15_H_24_	204	0.46 ± 0.03	MS, KI
30.24	1540	1545	α-Calacorene	C_15_H_20_	200	0.25 ± 0.02	MS, KI
30.81	1560	1565	β-Calacorene	C_15_H_20_	200	0.10 ± 0.01	MS, KI
32.54	1626	1628	1-*epi*-Cubenol	C_15_H_26_O	222	0.41 ± 0.03	MS, KI
32.91	1641	1646	δ-Cadinol	C_15_H_26_O	222	1.31 ± 0.09	MS, KI
33.23	1654	1654	α-Cadinol	C_15_H_26_O	222	1.39 ± 0.10	MS, KI
38.69	1898	1896	Rimuene	C_20_H_36_	276	1.21 ± 0.14	MS, KI
41.63	2047	2043	16-Kaurene	C_20_H_32_	272	12.43 ± 1.32	MS, KI
Sesquiterpene Hydrocarbons						69.35 ± 2.18	
Oxygenated Sesquiterpenes						3.10 ± 0.21	
Diterpene Hydrocarbons						13.64 ± 1.46	
Total Identified						86.09 ± 0.51	

^a^ RT: Retention time; ^b^ KI: Kovats index relative to *n*-alkanes (C7–C30) on a DB-5MS column; ^c^ rKI: reference Kovats index from other research [36]; ^d^ MS: NIST and Wiley libraries spectra and literature; KI: Kovats index.

**Table 2:** IC_50_ values of *A. dammara* leaf essential oil against mushroom tyrosinase.

Specimen	IC_50_ (μg/mL)	
	L-Tyrosine as the Substrate	L-DOPA as the Substrate
Leaf essential oil	- *	690.02 ± 18.85
Kojic acid **	2.46 ± 0.06	6.74 ± 0.09

*: >800 μg/mL; **: Positive control.

**Table 3:** Inhibitory effect of *A. dammara* leaf essential oil on melanogenesis of zebrafish embryos.

Specimen	Concentration (μg/mL)	Inhibition (%)
PTU	50	98.23 ± 1.01 ^d^
Arbutin	25	19.62 ± 9.10 ^a,b^
	50	21.49 ± 5.98 ^a,b^
Kojic acid	25	18.53 ± 3.56 ^a^
	50	20.44 ± 7.27 ^a,b^
Leaf essential oil	12.5	21.03 ± 10.02 ^a,b^
	25	37.36 ± 9.79 ^b,c^
	50	43.48 ± 7.30 ^c^

PTU: 1-Phenyl-2-thiourea. Different letters (a–d) in the table indicate significantly different inhibition values between specimens at the level of *p* < 0.05 according to the Scheffe’s test.

## Data Availability

The data are available are available from the corresponding author on reasonable request.
